# Causal Relationships Between the Gut Microbiota, Inflammatory Cytokines, and Amyotrophic Lateral Sclerosis: A Mendelian Randomization Analysis

**DOI:** 10.1002/brb3.70571

**Published:** 2025-05-18

**Authors:** Li Changqing, Yu Leying, Ma Caiyun, Wen Hebao, Han Laiguo, Zhao Xiaojiang

**Affiliations:** ^1^ Department of Physical Education and Arts Bengbu Medical University Bengbu China; ^2^ Anhui Engineering Research Center for Neural Regeneration Technology and Medical New Materials Bengbu Medical University Bengbu China

**Keywords:** Gut microbiota, Amyotrophic Lateral Sclerosis, inflammatory cytokines, Mendelian randomization

## Abstract

**Background:**

The relationship between gut microbiota (GM) and amyotrophic lateral sclerosis (ALS) is well‐documented. However, the causal nature of this association and the potential mediating role of inflammatory cytokines (ICs) have yet to be elucidated.

**Methods:**

We performed Mendelian randomization (MR) analyses utilizing data derived from genome‐wide association studies (GWAS) of GM, ICs, and ALS. Initially, we conducted bidirectional two‐sample MR analysis to determine the causal relationships between GM, ICs, and ALS. Subsequently, a two‐step MR mediation analysis was performed to investigate the role of ICs as mediators. The primary statistical approach was the inverse variance weighted (IVW) method.

**Results:**

Through MR analysis, we identified one positive causal relationship and three negative causal relationships between GM and ALS. There was one positive association and one negative association between ICs and ALS. In addition, ICs do not appear to mediate the pathway from GM to ALS.

**Conclusion:**

This study established a causal relationship between GM, ICs, and ALS, suggesting that ICs do not function as mediators in the pathway from GM to ALS. These findings provide new perspectives on potential ALS prevention and treatment strategies.

## Graphical abstract

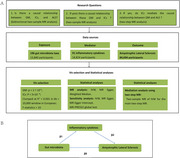

## 1 Introduction

Amyotrophic lateral sclerosis (ALS) is a heterogeneous neurodegenerative disease of obscure etiology that fundamentally affects motor neurons (MNS) in the motor cortex and spinal cord (Pribac et al. 2024). Like most neurodegenerative diseases, ALS usually starts with local muscle weakness and progresses to most muscles, including the diaphragm, and ultimately the patient dies of respiratory paralysis within 3–5 years (Alonso et al. 2009). In addition, ALS is somewhat heritable, with about 10% of ALS cases running within families (Taylor et al. 2016). The worldwide annual incidence of ALS varies between 0.26 and 23.46 cases per 100,000 individuals, and approximately 30,000 deaths are linked to ALS each year (Masrori and Van Damme 2020). Currently, two recognized therapeutic drugs, riluzole (glutamate antagonist) and edaravone (free radical scavenger), have been classified as disease‐relieving drugs, but their effectiveness is limited (Bensimon et al. 1994; Rothstein 2017).

Gut microbiota (GM), a diverse microbial community comprising bacteria, fungi, and viruses, inhabits the host's gastrointestinal tract (Lozupone et al. 2012). Emerging evidence suggests that gastrointestinal dysfunction may contribute to neurodegenerative diseases by affecting the microbiota‐gut‐brain axis (MGBA). For instance, Parkinson's disease, Alzheimer's disease, and Huntington's disease (Pellegrini et al. 2018; Wang et al. 2021). Recent studies indicate that metabolites from the gastrointestinal tract, associated with the GM group, can cross the blood–brain barrier and contribute to ALS pathogenesis (Cryan et al. 2020; Niccolai et al. 2021). Moreover, the translocation or abundance change of GM is inextricably related to the influences, course, treatment options, and prognosis of ALS. (Blacher et al. 2019; Boddy et al. 2021; McCombe et al. 2019).

Inflammatory cytokines (ICs) are small peptides produced and released by both immune and non‐immune cells. These cytokines have a variety of biological activities (Kany et al. 2019). Although the exact cause of ALS is unknown, it has been found that abnormal expression of ICs has been implicated in neuronal survival and function, thereby promoting disease progression (Ravnik‐Glavač et al. 2022; Yusuf et al. 2022). Researchers have identified a number of cytokines, including many interleukins and immune cells, in cerebrospinal fluid, plasma, or serum, for instance regulatory cells (Lu et al. 2016). These results suggest that both GM and ICs can influence the development of ALS to some extent.

Mendelian randomization (MR) studies provide a novel approach to assess causality between exposures and outcomes by combining data collected from GWAS with genetic variations to generate exposure‐related instrumental variables (IVs) (Burgess et al. 2015). MR analysis decreases the likelihood of confounders, reverse causation, or other biases that affect the accuracy of results obtained in traditional observational studies (Cong et al. 2022). In line with these premises, we posit that ICs may mediate the influence of GM on ALS risk. MR analysis was applied for determining causal relationships of GM and ICs with ALS. Bidirectional MR and mediation analyses were carried out using GWAS data on human GM, ICs, and ALS to examine the correlations between them.

## 2 Materials and Methods

### 2.1 Study Design

**FIGURE 1:**
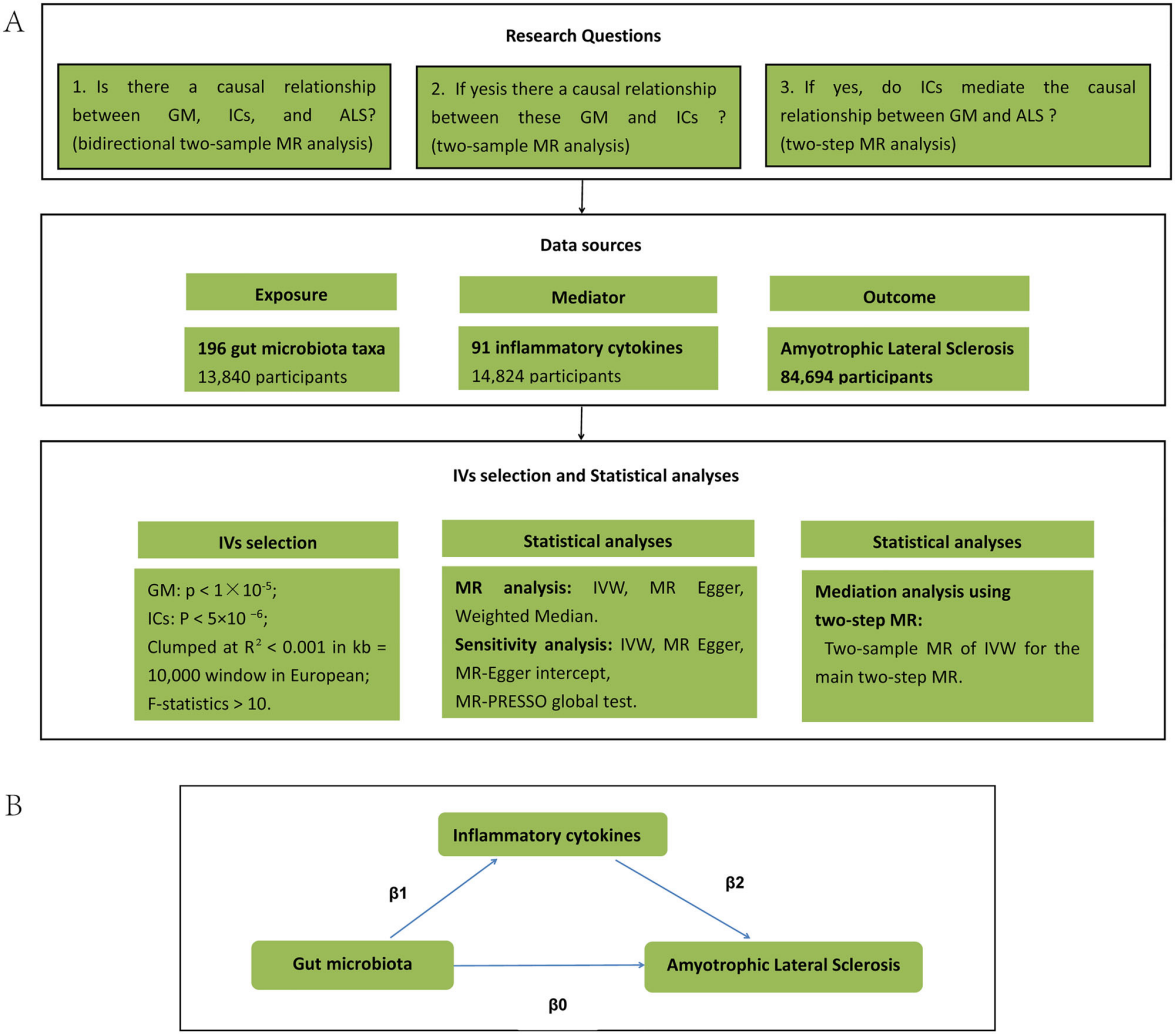
**Schematic representation of the study design and the two‐step Mendelian randomization analysis**. Panel A illustrates the study design, while Panel B depicts the diagram of the two‐step Mendelian randomization process. β0 was the total effect of GM on ALS; β1 represents the causal effects of GM on ICs; β2 represents the causal effects of ICs on ALS. The mediation effect was computed as the product of “β1” and “β2” (β1 × β1), and the mediation proportion was calculated as the ratio of the mediation effect product to total effects [(β1 × β1)/β0]. Abbreviations: GM = gut microbiota; ICs = inflammatory cytokines; MVMR = multivariable MR; IVW = inverse variance weighted; IVs = instrumental variable; ALS = amyotrophic lateral sclerosis.

We investigated the genetic association of GM and ICs with ALS by using publicly accessible GWAS data. First, two‐sample MR (TSMR) analysis was conducted to reveal bidirectional causality between GM, ICs, and ALS (Figure 1A). Subsequently, we used the multivariate MR (MVMR) method for quantitative analysis. We also explored whether ICs function as mediators in the association pathway between the GM and ALS (Figure 1B). We performed the MR analysis by following the STROBE‐MR checklist. Single nucleotide polymorphisms (SNPs) were defined as IVs. The following core assumptions were considered for the MR analysis: (1) IVs should exhibit an association with exposures; (2) no relationship should exist between IVs and confounders related to exposures and outcomes; and (3) IVs should affect the outcomes only through exposures.

### 2.2 Data Sources

In this study, GM and ICs were defined as exposure factors, while ALS was defined as the outcome factor (Figure 1A). We obtained GM data from extensive analyses conducted by the MiBioGen consortium. The dataset integrates genetic information from 24 cohorts, including 18,340 individuals from 11 countries, mostly participants of European descent (Kurilshikov et al. 2021). This systematic collation process resulted in a dataset containing 196 bacterial taxonomic units, providing a comprehensive basis for in‐depth analyses. Information on 91 ICs was gathered from GWAS data with the Olink Target Inflammation panel spanning 11 cohorts, including 14,824 people of European origin (Zhao et al. 2023). Data for the results section were obtained from publicly available Europe‐based ALS genotyping GWAS aggregated statistics, which derived 10,181,076 SNPs in 22,040 cases and 62,644 controls (Iacoangeli et al. 2020).

### 2.3 IVs Selection and Data Harmonization

We implemented a series of criteria to screen for eligible genetic variants. Initially, we planned to select IVs with a significance threshold of *p* < 5 × 10^−8^ to screen for GM and ICs. Due to the very low number of eligible IVs, based on previous studies, we used looser statistical thresholds. Ultimately, we applied significance thresholds of *p* < 1 × 10^−5^ to screen for IVs associated with GM and *p* < 5 × 10^−6^ to screen for IVs associated with ICs. Subsequently, based on linkage disequilibrium (LD) (*r* ^2^ = 0.001, kb = 10000), we adhered to the hypothesis of MR and excluded palindromic SNPs to reduce the influence of alleles on outcomes. The IVs' robustness was assessed using the *F*‐statistic *F* = *β* ^2^/SE^2^, in order to avoid weak instrumental bias (**Sun et al**. 2018 **)**.

### 2.4 Statistical Analyses

First, we performed comprehensive bidirectional TSMR analyses to assess the bidirectional causality between GM, ICs, and ALS. We used various methods in our analysis, primarily the IVW approach, with MR‐Egger and weighted median as secondary methods. MR analysis results were shown as odds ratios (OR) with 95% confidence intervals (95% CI). A *p* value of < 0.05 for the IVW approach was considered statistically significant, and the difference was statistically significant when the other two methods pointed in the same direction as the IVW approach. False discovery rate (FDR) correction was applied. Results were considered statistically significant when FDR < 0.1 and *p* < 0.05 but suggestive of an association when FDR ≥ 0.1 and *p* < 0.05. Second, we performed mediation analysis using a two‐step MR study to determine the role of ICs in mediating the relationship between GM and ALS.

### 2.5 Sensitivity Analysis

We employed a variety of methods to ensure the study's robustness and reliability. We used Cochran's *Q* statistic to test for heterogeneity in SNPs. A *p* value < 0.05 indicated significant heterogeneity, and we employed a random effects approach if heterogeneity was present. In addition, to detect potential horizontal pleiotropy effects, we utilized the MR‐Egger regression intercept and the MR‐PRESSO global test. A significance threshold of a *p* value < 0.05 suggests the presence of horizontal pleiotropy, while non‐significance indicates its absence. Analyses were conducted with R software (v4.3.2).

## 3 Result

### 3.1 Selection of IVs

Through comprehensive screening and analysis, we identified four distinct GMs and five ICs that exhibit potential causal relationships with ALS. The *F*‐statistics for the selected IVs in our study all surpass the threshold of 10, indicating no presence of weak IVs. Comprehensive findings are available in Tables S1 and S2.

### 3.2 Effects of GM on ALS

**FIGURE 2:**
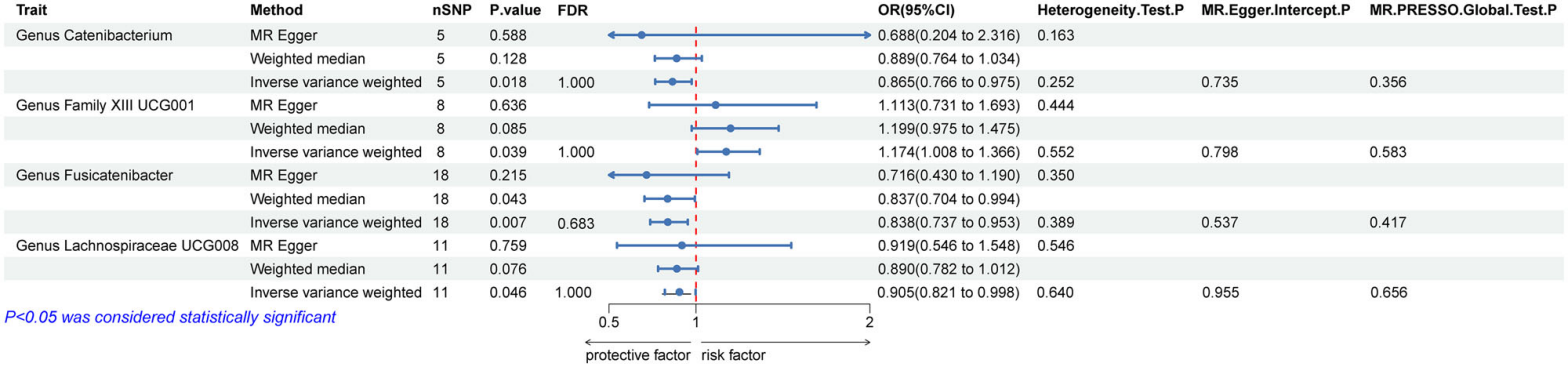
**Estimates from MR regarding the relationship between GM and ALS**. Abbreviations: GM = gut microbiota; nSNP = number of single‐nucleotide polymorphism; ALS = amyotrophic lateral sclerosis; FDR = false discovery rate.

When evaluating the causal association between GM and ALS, we identified statistical associations between four bacterial taxa and ALS risk (Figure 2). Specifically, genus family XIII UCG001 (OR = 1.174, 95% CI = 1.008–1.366, *p* = 0.039, FDR = 1.000) was positively correlated with ALS, indicating that genetic prediction of GM increased ALS risk; genus *Catenibacterium* (OR = 0.865, 95% CI = 0.766–0.975, *p* = 0.018, FDR = 1.000), genus *Fusicatenibacter* (OR = 0.838, 95% CI = 0.737–0.953, *p* = 0.007, FDR = 0.683), and genus *Lachnospiraceae* UCG008 (OR = 0.905, 95% CI = 0.821–0.998, *p* = 0.046, FDR = 1.000) were negatively correlated with ALS. These findings imply that three GM types may reduce the risk of ALS. However, following the application of the FDR correction, the results, while indicative of a potential association, did not reach statistical significance.

### 3.3 Effects of ICs on ALS

**FIGURE 3:**
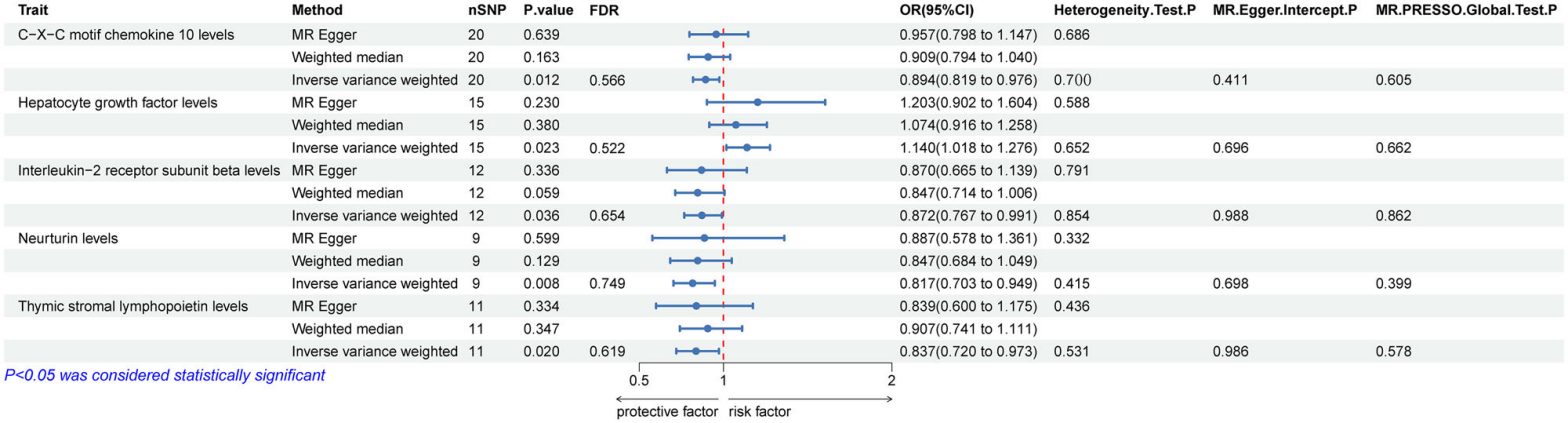
**Estimates from MR regarding the relationship between ICs and ALS**. Abbreviations: ICs = inflammatory cytokines; nSNP = number of single‐nucleotide polymorphisms; ALS = amyotrophic lateral sclerosis; FDR = false discovery rate.

There were five types of ICs causally associated with ALS in the IVW method (Figure 3). Among them, hepatocyte growth factor (HGF) levels (OR = 1.140, 95% CI = 1.018–1.276, *p* = 0.023, FDR = 0.552) were positively correlated with ALS, indicating that genetic prediction of ICS increased ALS risk. C–X–C motif chemokine 10 levels (OR = 0.894, 95% CI = 0.819–0.976, *p* = 0.012, FDR = 0.566), interleukin−2 receptor subunit beta levels (OR = 0.872, 95% CI = 0.767–0.991, *p* = 0.036, FDR = 0.654), neurturin levels (OR = 0.817, 95% CI = 0.703–0.949, *p* = 0.008, FDR = 0.749), and thymic stromal lymphopoietin levels (OR = 0.837, 95% CI = 0.720–0.973, *p* = 0.020, FDR = 0.619) were negatively correlated with ALS. It is suggested that genetically forecasting these four ICs is associated with reduced risk of ALS. However, after the FDR correction, the results hinted at an association but were not statistically significant.

### 3.4 Reverse MR Analysis and Mediation Analysis

For reverse MR analysis, as demonstrated in Tables S3 and S4, we found five causal associations between ALS and GM, and there were three associations between ALS and ICs. In this study, both GM and ICs had a causal effect on ALS. ICs seem to mediate the GM‐ALS pathway. One of the conditions for the mediating role is that GM is significantly correlated with ICs. However, the results of two‐step MR analyses demonstrated no causal link between ALS‐related GM and ICs (Table S5), suggesting that ICs do not function as intermediaries in the pathway between GM and ALS.

### 3.5 Sensitivity Analysis

Multiple sensitivity analyses were conducted to assess and adjust for potential pleiotropy in estimating causal effects. No notable heterogeneity was detected in this MR Analysis (Cochran's *Q* > 0.05). Furthermore, there was no evidence of pleiotropy found in the MR‐Egger regression intercept test (*p* > 0.05) and the MR‐PRESSO global test (*p* > 0.05) (Figures 2 and 3).

## 4 Discussion

Our results identified one positive and three negative associations between GM and ALS. In addition, our analysis identified one positive and four negative correlations between ICS and ALS. No evidence supports ICS as a mediator in the GM to ALS pathway.

Our research shows that *Catenibacterium* and *Fusicatenibacter* are beneficial factors in ALS. Ning et al. (2022)’s study also suggests that the decrease in *Fusicatenibacter* and *Catenibacterium* and the increase in *Lachnospira* in patients may be related to the high risk of ALS. It is known that the decrease of *Catenibacterium*, which produces short‐chain fatty acid (SCFA) through dietary fiber fermentation, can lead to the decrease of SCFA levels. SCFA is essential for controlling inflammation and enhancing muscle protein synthesis (Song et al. 2024). In addition, *Lachnospiraceae* can also produce SCFA (Nishiwaki et al. 2022). In recent years, the beneficial effects of trehalose on ALS have been discovered (Calvete‐Torre et al. 2024). Research has revealed that certain beneficial bacteria are markedly different in ALS patients; for instance, patients with ALS have decreased *Ruminococcus*, *Prevotella*, *Erysipelotrichaceae*, *Fusicatenibacter*, and *Roseburia ininalis* (Chen et al. 2023). In addition, studies indicate that compared to ALS patients, healthy controls showed concentrations of Erysipelotrichaceae_UCG‐003, *Fusicatenibacter*, and *Subdoligranulum* (Martin et al. 2022). These findings all suggest a negative correlation between *Fusicatenibacter* and ALS, which is consistent with our experimental findings. The relative abundance levels of two bacteria that produce butyrate, *Anaerostipes* and *Lachnospiraceae*, in the gut of ALS patients were significantly lower than in healthy controls (Fang et al. 2016). At the same time, a decrease in *Lachnospiraceae* was also observed in ALS animal models, and changes in intestinal flora occurred early in ALS, even before the deterioration of motor capacity (Zhang et al. 2021). Butyric acid‐producing bacteria, for instance, *Lachnospiraceae* and *Pseudobutyrivibrio* are associated with an increase in L‐leucine and asparagine (Zhufeng et al. 2022). Furthermore, research has indicated that treatment with butyrate, alongside antibiotic therapy, can help restore the function of the intestinal mucosa and rectify imbalances in GM in mice suffering from ALS (Zhang et al. 2021). Our results also show the beneficial effects of *Lachnospiraceae* on ALS.

Interleukin‐2‐mediated inflammatory response is proposed to contribute to ALS development (Lu et al. 2016). In addition, it has been noted that Interleukin‐2 is associated with a lower risk of ALS (Liu et al. 2023). This may be due to differences in the number of samples and selection of materials. Our study suggests that neurturin levels are a beneficial factor in ALS. One study identified the myokine neurturin as a key component of PGC‐1α1‐mediated neuromuscular recruitment to muscle (Mills et al. 2018). HGF is one of the most potent pro‐survival factors for MNS and is a promising drug for clinical application in the treatment of ALS (Funakoshi et al. 2007). If sufficient HGF is supplied to MNS and astrocytes in the spinal cord, it may be possible to reduce the depletion of endogenous HGF during the disease process (Yamamoto et al. 2008). This suggests that HGF is a beneficial factor in ALS, contrary to our findings, which may be due to differences in sample size and selection.

The study has several advantages. First, we investigated the relationship between GM and ALS by the MR method and whether ICs acted as intermediaries. Second, we make use of large‐scale GWAS data, and this extensive dataset ensures strong statistical power and produces rich results. Lastly, we employed FDR correction for multiple comparisons. The study does, however, have certain shortcomings. First, the majority of these subjects were European, which may not accurately reflect the genetic and lifestyle diversity that affects the GM of different populations. Second, based on existing GWAS, the most specific classification level of transgenes in our study is genus, and therefore at a lower classification level. Third, although we explored the mediating role of ICs between different intestinal flora abundance and ALS, considering that ICs do not act as mediators, the mechanism of how intestinal flora affects the pathogenesis of ALS remains to be studied. Fourth, it is also important to recognize that our findings did not survive the strict threshold of the FDR correction. However, applying such a stringent correction has the potential to obscure biologically meaningful findings. Fifth, we selected the IVs for GM at *p* < 1.0 × 10^−5^ and ICs at *p* < 1.0 × 10^−5^ which were larger than the traditional genome‐wide significance level (*p* < 5 × 10^−8^) to obtain sufficient IVs. If an exposure has weak IVs in previous studies, the threshold can be relaxed to avoid missing causal variables. Finally, GM composition and ICs levels in the human body are dynamic indicators; however, MR studies do not capture changes in them. To gain a more comprehensive understanding of the causal link between GM, ICs, and ALS, long‐term and longitudinal research is needed to observe their evolution.

## 5 Conclusions

Our study offers genetic evidence indicating a connection between GM, ICs, and ALS. Specifically, we identified one positive and three negative correlations between GM and ALS, as well as one positive and four negative correlations between ICs and ALS. Furthermore, reverse MR analysis revealed five causal associations between ALS and GM and three associations between ALS and ICs. Notably, ICs do not seem to function as a mediating factor in the pathway linking GM to ALS.

## Author Contributions

**Li Changqing**: data curation, writing – review and editing, funding acquisition, writing – original draft. **Yu Leying**: software, methodology, writing – original draft. **Ma Caiyun**: visualization. **Wen Hebao**: validation. **Han Laiguo**: formal analysis. **Zhao Xiaojiang**: project administration, funding acquisition, data curation, writing – review and editing.

## Ethics Statement

Participants consented to the GWAS as per the original protocols, and the original authors were responsible for obtaining all ethical approvals.

## Conflicts of Interest

The authors declare no conflicts of interest.

### Peer Review

The peer review history for this article is available at https://publons.com/publon/10.1002/brb3.70571.

## Supporting information



Table S1 Used IVs for 196 gut microbiota taxa from MiBioGen.
Table S2 Used IVs for 91 inflammatory cytokines taxa from GWAS Catalog.
Table S3 MR results of amyotrophic lateral sclerosis on gut microbiota
Table S4 MR results of amyotrophic lateral sclerosison inflammatory cytokines
Table S5 MR results of gut microbiota on inflammatory cytokines.

## Data Availability

The datasets that were analyzed in this study are not publicly available. The corresponding author can be contacted for further inquiries.
